# A novel algorithm for a precise analysis of subchondral bone alterations

**DOI:** 10.1038/srep32982

**Published:** 2016-09-06

**Authors:** Liang Gao, Patrick Orth, Lars K. H. Goebel, Magali Cucchiarini, Henning Madry

**Affiliations:** 1 Center of Experimental Orthopaedics, Saarland University, Homburg, Germany; 2 Department of Orthopaedic Surgery, Saarland University Medical Center, Homburg, Germany

## Abstract

Subchondral bone alterations are emerging as considerable clinical problems associated with articular cartilage repair. Their analysis exposes a pattern of variable changes, including intra-lesional osteophytes, residual microfracture holes, peri-hole bone resorption, and subchondral bone cysts. A precise distinction between them is becoming increasingly important. Here, we present a tailored algorithm based on continuous data to analyse subchondral bone changes using micro-CT images, allowing for a clear definition of each entity. We evaluated this algorithm using data sets originating from two large animal models of osteochondral repair. Intra-lesional osteophytes were detected in 3 of 10 defects in the minipig and in 4 of 5 defects in the sheep model. Peri-hole bone resorption was found in 22 of 30 microfracture holes in the minipig and in 17 of 30 microfracture holes in the sheep model. Subchondral bone cysts appeared in 1 microfracture hole in the minipig and in 5 microfracture holes in the sheep model (n = 30 holes each). Calculation of inter-rater agreement (90% agreement) and Cohen’s kappa (kappa = 0.874) revealed that the novel algorithm is highly reliable, reproducible, and valid. Comparison analysis with the best existing semi-quantitative evaluation method was also performed, supporting the enhanced precision of this algorithm.

The subchondral bone provides a functional support for the articular cartilage, the gliding tissue that covers the articulating ends of bones. It is often involved in defects of the articular cartilage that are difficult to treat^1^,^2^,^3^,^4^. Subchondral bone alterations are emerging as a considerable clinical problem associated with different articular cartilage repair techniques^4^. Their analysis reveals a pattern of variable changes such as intra-lesional osteophytes, residual holes originating from marrow stimulation procedures, peri-hole bone resorption, and subchondral bone cysts.

A significant level of subchondral bone changes is found after marrow stimulation for cartilage repair^5^. Here, multiple perforations of the subchondral bone plate are introduced with awls^4^ or burrs^6^, allowing for the access of pluripotent mesenchymal stem cells from the marrow cavity to the site of the cartilage defect^7^,^8^. Despite clinical short- and mid-term success^1^,^9^, subchondral bone changes are thought to influence the long-term outcome of this procedure^4^,^5^,^8^,^10^. For example, subchondral bone cysts or intra-lesional osteophytes^5^ were described in up to 33% of patients treated with marrow stimulation^3^,^7^,^9^,^11^.

Similar patterns of subchondral bone changes around the original holes were also observed in preclinical studies using various translational animal models of microfracture treatment^12^,^13^,^14^,^15^,^16^,^17^. Previous translational studies have attempted to evaluate these postoperative morphological changes of the subchondral bone by histology^12^,^15^,^16^ and conventional radiography^13^,^14^,^17^. Yet, a precise distinction especially between residual microfracture holes, peri-hole bone resorption, and cyst formation is often difficult to make when applying these methods.

High-resolution micro-computed tomography (micro-CT) allows for a quantitative evaluation of the subchondral microarchitecture in a three-dimensional (3D) and non-invasive manner, overcoming the limitations of histology and conventional radiographic modalities. Chen *et al*. recently proposed a micro-CT-based scoring system to estimate subchondral bone changes in rabbits after microfracture treatment^12^. However, the categorical scoring system described by the authors relies on subjective evaluations and yields semi-quantitative information. Consequently, the obtained data do not represent the actual measurements on a continuous scale, hindering the comparison of studies with different designs. A detailed and reproducible algorithm allowing for a sufficient instructional analysis of subchondral bone changes after microfracture treatment in micro-CT images has, to our very best knowledge, not been proposed to date.

In our study, we chose the microfracture technique to investigate subchondral bone changes as it represents the most commonly used marrow stimulation method. Our strategy was (1) to review the analytic methods described to examine the major types of subchondral bone changes after microfracture treatment in experimental animal models in PubMed records, (2) to develop a novel algorithm for the analysis of these subchondral bone changes based on micro-CT images, allowing for a specific postoperative distinction between intra-lesional osteophytes, residual microfracture holes, peri-hole bone resorption, and subchondral cyst formation, (3) to evaluate this algorithm in datasets from two large animal models, and (4) to compare it with the best existing evaluation method.

## Results

### Identification of current analytic methods to examine subchondral bone changes

A total of 7 articles were identified in the PubMed database that provide analytic methods to examine the major types of subchondral bone alterations following microfracture treatment in preclinical animal models such as the formation of intra-lesional osteophytes (n = 2 articles), residual microfracture holes (n = 3), peri-hole bone resorption (n = 3), and subchondral bone cysts (n = 4) (Supplementary Fig. 1). Most of these attempts to evaluate such morphological changes were performed by histology and radiography, including plain radiography, magnetic resonance imaging (MRI), and micro-CT (Table 1).

### Algorithm for the analysis of subchondral bone changes in micro-CT images

**Figure 1:**
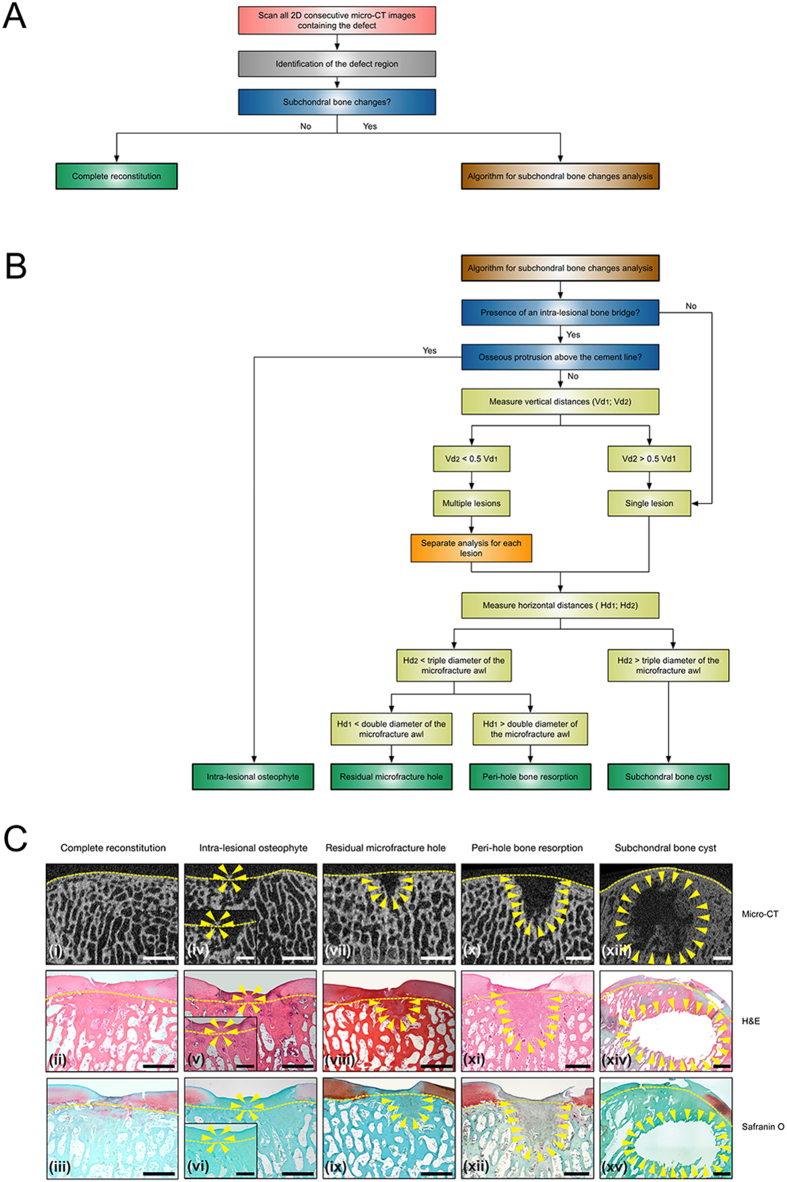
Micro-CT based algorithm for the analysis of the subchondral bone changes following microfracture treatment. (**A**) General workflow for analysis of subchondral bone changes based on consecutive 2D micro-CT images containing the entire subchondral bone defect. “Complete reconstitution” is reported only if the defect region resembles a normal subchondral bone structure. For a defect showing subchondral bone alterations, the second part of the algorithm has to be applied to evaluate each lesion within the defect. (**B**) Algorithm for analysis of the four patterns of subchondral bone changes. Of note, for a defect containing multiple lesions, each lesion should be evaluated separately, and the outcome of each lesion should also be reported respectively. For additional explanation of Vd_1_, Vd_2_, Hd_1_, and Hd_2_, please refer to Fig. 2. (**C**) Representative micro-CT, haematoxylin and eosin (H&E), and safranin orange/fast green (safranin O) stained images of the five possible outcomes of the present algorithm applied in the minipig (i–xii) and sheep (xiii–xv) models. Insets show the higher magnification images of an intra-lesional osteophyte (iv–vi). The yellow dashed lines denote the projected cement line, and the yellow triangles define the contour of the specific subchondral bone alteration. Subchondral bone cysts were only observed in the sheep model, while the other four patterns were detected in both models. Scale bars, 10 mm (i–xv) and 5 mm (insets; iv–vi).

To standardise the analysis of subchondral bone changes, we developed a micro-CT image based algorithm consisting of two parts. In the first part (Fig. 1A), all two-dimensional (2D) consecutive micro-CT images containing the entire subchondral defect must be scanned to identify the defect region. Then the presence of subchondral bone changes should be confirmed. If the subchondral bone of the entire defect treated with microfracture is completely restored, the defect will be classified as “complete reconstitution”. If not, the second part of the algorithm should be applied (Fig. 1B).

**Figure 2:**
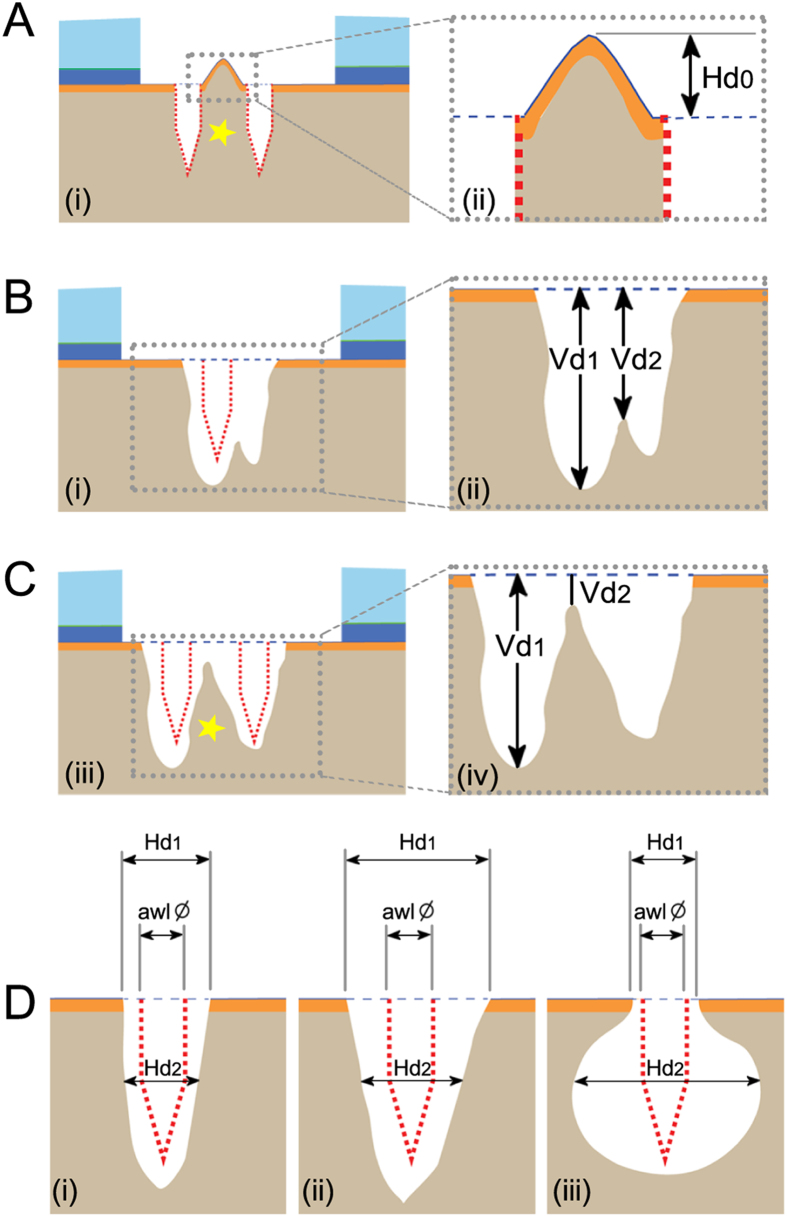
Identification of intra-lesional osteophyte, residual microfracture hole, peri-hole bone resorption, and subchondral bone cyst. (**A**) An intra-lesional osteophyte is defined as an osseous overgrowth protruding above the projected cement line. The maximal osteophyte height (H_0_) is measured as the perpendicular height from the top to the projected cement line. (**B**) Vd_1_ and Vd_2_ are the vertical distances from the projected cement line to the base of the defect and to the top of the suspected bone bridge respectively. A single microfracture hole is defined without the intra-lesional bone bridge (Vd_2_ > 0.5 Vd_1_), indicating that the subchondral bone change is derived from bone alteration surrounding the single hole. (**C**) Two microfracture holes are defined with a persistent bone bridge (Vd_2_ < 0.5 Vd_1_), necessitating the separate analysis of the pattern of subchondral bone change for each hole. (**D**) Discrimination between residual microfracture hole (i), peri-hole bone resorption (ii), and subchondral bone cyst (iii) using the relative horizontal diameters (Hd_1_: the horizontal diameters at the level of the opening of the hole; Hd_2_: the horizontal diameter at the half of the penetration depth). Using the diameter of the microfracture awl as reference, a residual microfracture hole (i) is defined with a small opening (Hd_1_ < 2 awl diameter; Hd_2_ < 3 awl diameter), and peri-hole bone resorption (ii) is defined with a large opening (Hd_1_ > 2 awl diameter; Hd_2_ < 3 awl diameter). (iii) A subchondral bone cyst is defined when Hd_2_ is larger than the triple diameter of the microfracture awl (Hd_2_ > 3 awl diameter), regardless of Hd_1_. The red dotted lines show the original location of the microfracture holes and the blue dotted lines indicate the projected cement line. The yellow asterisks in (**A,C**) indicate the bone bridge between two microfracture holes. Ø: diameter.

When applying this second part (Fig. 1B), the presence of any bone bridge between microfracture holes must first be confirmed. An osseous upward overgrowth of such a bone bridge is defined as an intra-lesional osteophyte (Fig. 2A). Second, the number of microfracture holes within the osteochondral region is determined according to the relative height of the bone bridge (Fig. 2B,C). Of note, for a defect containing multiple lesions, each lesion should be evaluated individually. Third, for each lesion, discrimination between residual microfracture holes, peri-hole bone resorption, and subchondral bone cysts should be performed based on the suggested thresholds (Fig. 2D).

#### Definition of an intra-lesional osteophyte

The intra-lesional bone bridge serves as the first evaluation index of the algorithm. By definition, a bone bridge only exists when the micro-CT image shows two microfracture holes. This bony structure above the cement line is termed intra-lesional osteophyte if a bone bridge protrudes above the projected cement line (Fig. 2A)^18^. The location of such an intra-lesional osteophyte is either central (between two microfracture holes) or peripheral (between microfracture hole and the defect edge)^18^. The maximal height of each osteophyte (H_0_) can be measured as the vertical distance from the proximal end of the osteophyte to the projected cement line (Fig. 2Aii). The maximal width and two-dimensional (2D) area of each osteophyte can also be reported.

#### Definition of the relative bone bridge height as a means to differentiate between one or multiple holes

The definition of the relative bone bridge height defines the number of visible microfracture holes. This bone bridge is usually located below the projected cement line. Such a beneath located bone bridge can, however, either result from bone resorption around a single microfracture hole or represent the remnant of a bone bridge between two original microfracture holes. In order to identify such a suspected bone bridge, it is proposed to first calculate the relative height of the suspected bone bridge. In micro-CT images, the projected cement line is used as the apical reference line and the vertical distance (Vd) is measured from this reference line to the base of defect (Vd_1_) and to the top of the suspected bone bridge (Vd_2_) (Fig. 2Bii). If the vertical distance from the projected cement line to the top of the suspected bone bridge is larger than half of the distance to the base of the defect (Vd_2_ > 0.5 Vd_1_), this subchondral bone alteration is considered to originate from a single microfracture hole with surrounding bone erosion (Fig. 2Bi). On the contrary, if the vertical distance from the projected cement line to the top of a suspected bone bridge is less than half of the distance to the base of the defect (Vd_2_ < 0.5 Vd_1_), a persistent bone bridge is present. In this case, each of the two microfracture holes beside this persistent bone bridge requires separate analysis (Fig. 2C).

#### Discrimination between residual microfracture holes, bone resorption, and subchondral bone cysts

To distinguish between residual microfracture holes, bone resorption, and subchondral bone cysts, the horizontal diameters (Hd) at the opening of each microfracture hole (Hd_1_) and the maximum horizontal diameter at half of the penetration depth (Hd_2_) are measured (Fig. 2D). In order to allow for a comparison between holes, Hd_2_ is measured in parallel to Hd_1_ at 50% of the penetration depth of the microfracture awl. If Hd_2_ is less than the triple diameter of the microfracture awl, differentiation between residual microfracture hole and peri-hole bone resorption is mandatory.

Using the diameter of the microfracture awl as the reference standard, a residual microfracture hole is identified when the horizontal diameter of the opening of the hole (Hd_1_) is less than the double diameter of the microfracture awl (Fig. 2Di).

Peri-hole bone resorption is present when Hd_1_ is larger than the double diameter of the microfracture awl (Fig. 2Dii).

Per definition, a subchondral bone cyst possesses a horizontal diameter (at 50% of the penetration depth; Hd_2_) larger than the triple diameter of the microfracture awl (Fig. 2Diii), irrespective of Hd_1_.

The maximal depth of each hole and cyst can be measured from the projected cement line to the base of the defect, and the maximal size may be reported as the 2D area of the empty cavity below the projected cement line and within the subchondral bone^18^.

### Evaluation of the algorithm for the analysis of subchondral bone changes in two large animal models

**Figure 3:**
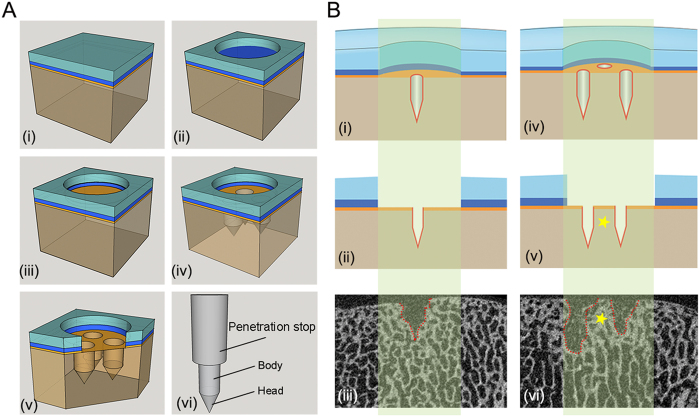
Schematic of microfracture procedure in animal models and micro-CT image patterns of the osteochondral unit in the minipig model. (**A**) Creation of a full-thickness chondral defect and treatment with the microfracture procedure. (i) The osteochondral unit is composed of the articular cartilage and the underlying subchondral bone. The subchondral bone is formed by the subchondral bone plate and the subarticular spongiosa. (ii) Removal of the hyaline cartilage to generate a circular chondral defect. (iii) Meticulous debridement of the calcified cartilage layer. (iv) Penetration of the subchondral bone plate with a microfracture awl to introduce three microfracture holes. (v) Detailed structure of the osteochondral unit following the microfracture treatment. (vi) Structure of the microfracture awl applied in both animal studies. The distal end has a conical head and a cylindrical body (1.2 mm diameter) and the proximal penetration stop standardizes the penetration depth. (**B**) Two micro-CT image patterns of the microfracture holes within defects of the minipig model. The osteochondral specimens are sectioned along their longitudinal axis by micro-CT. The light green-shaded boxes indicate the vertical cross section of the defect area. A section crossing a single microfracture hole (i,ii) corresponds to a micro-CT image (iii) displaying a single hole within the subchondral bone and a section crossing two microfracture holes (iv,v) corresponds to a micro-CT image (vi) showing two holes. The yellow asterisks (v,vi) indicate the inter-hole (intra-lesional) bone bridge. Dotted lines in (iii,vi) show the border of the residual microfracture holes. Colors of the osteochondral unit are for illustration purposes only including articular cartilage (light blue), calcified cartilage layer (dark blue), subchondral bone plate (orange), and subarticular spongiosa (brown).

**Figure 4:**
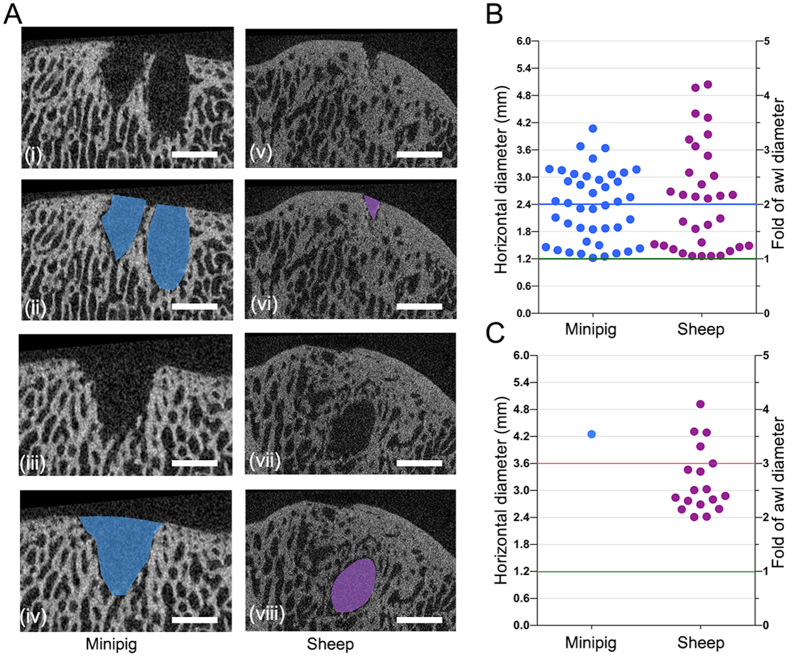
Representative micro-CT images of a single defect possessing multiple patterns of subchondral bone changes and the quantitation of the subchondral bone changes applying different thresholds of the algorithm. (**A**) Representative micro-CT images from defects of minipigs and sheep. Two suspected residual holes (i) and bone resorption (iii) were observed in the same minipig defect, and a suspected residual hole (v) and a cyst-like lesion (vii) were detected in the same sheep defect. (**B**) Quantitative estimation of residual microfracture holes and peri-hole bone resorption applying the algorithm with different thresholds in the minipig and sheep models. (**C**) Quantitative estimation of subchondral bone cysts applying the algorithm with different thresholds in the two models. The green horizontal lines (**B,C**) indicate the diameter of the microfracture awl as the reference standard. The red lines (**B,C**) indicate the triple diameter of the microfracture awl, as the suggested lower threshold value for a subchondral bone cyst. The blue horizontal line (**B**) indicates the double diameter of the microfracture awl, as the suggested lower threshold value of the peri-hole bone resorption. Scale bars, 2.0 mm.

**Figure 5:**
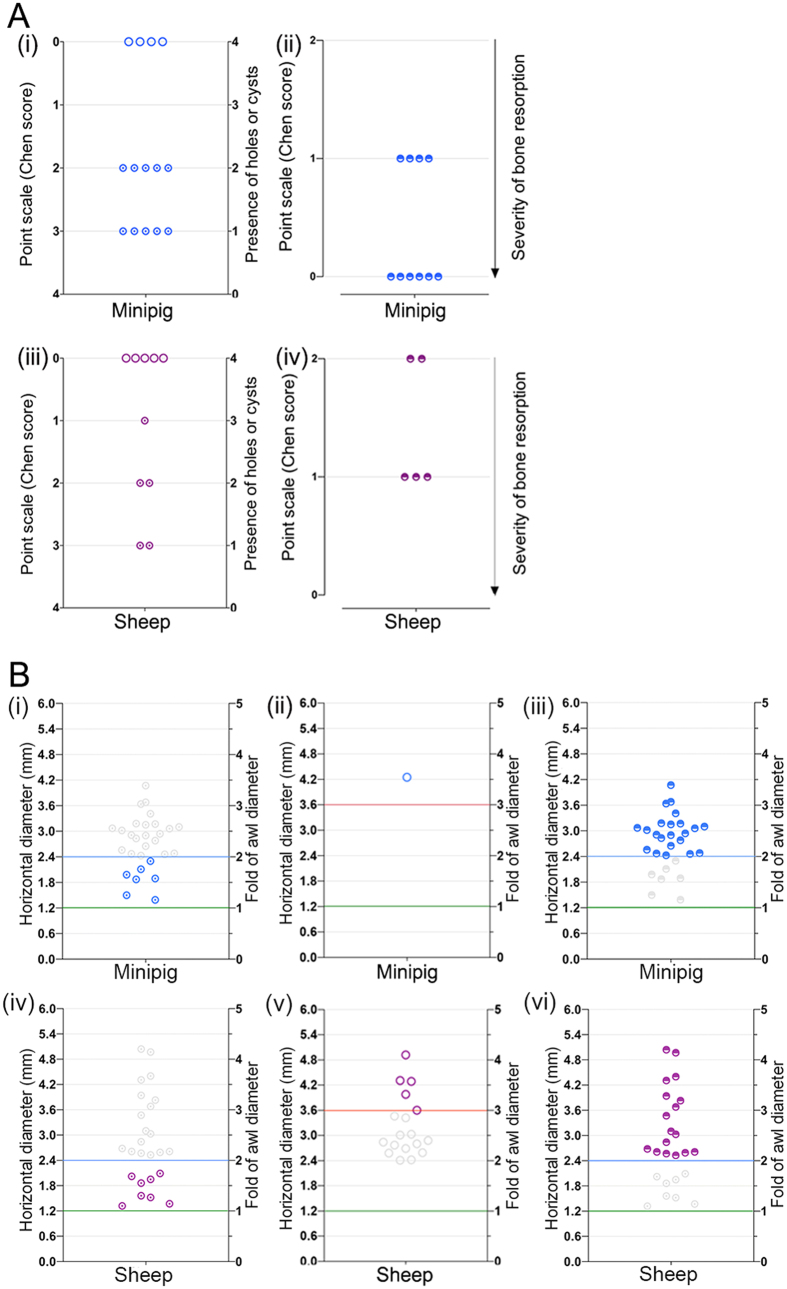
Comparison of the results of the Chen score and the present algorithm for analysing the subchondral bone changes in the two animal models. (**A**) Results of the Chen score: (i) residual microfracture holes and subchondral bone cysts in minipigs, (ii) bone resorption in minipigs, (iii) residual microfracture holes and subchondral bone cysts in sheep, and (iv) bone resorption in sheep. (**B**) Results of the present algorithm: (i) residual holes in minipigs, (ii) subchondral bone cyst in minipigs, (iii) peri-hole bone resorption in minipigs, (iv) residual holes in sheep, (v) subchondral bone cysts in sheep, and (vi) peri-hole bone resorption in sheep. Compared with the Chen categorical score, the present algorithm enables an objective quantitation and discrimination of residual microfracture holes, peri-hole bone resorption, and subchondral bone cysts in both minipig and sheep models. The grey symbols in (**B**) indicate the eliminated subchondral bone changes according to the proposed cut-off values of the present algorithm.

This algorithm for the analysis of subchondral bone changes was next evaluated in micro-CT datasets obtained from both the minipig and sheep models. All defects from these studies were full-thickness chondral defects located in the trochlea of the distal femur and were always treated with the same custom-made microfracture awl with a 1.2 mm diameter trihedral tip (Fig. 3Avi). Ten defects in minipigs with 3 microfracture holes per defect (Fig. 3A) and 5 defects in sheep with 6 microfracture holes per defect were randomly selected for the analysis with the present algorithm. Representative micro-CT images from both animal models are shown in Fig. 4A.

#### Analysis of the threshold for defining peri-hole bone resorption

When applying the standard conditions regarding Hd_1_ described above for the definition of peri-hole bone resorption (Hd_**1**_larger than the double diameter of the microfracture awl), this pathological condition was detected in 22 out of 30 microfracture holes (73%) in the minipig model and 17 out of 30 microfracture holes (57%) in the sheep model (Fig. 4B). Interestingly, when elevating the threshold for peri-hole bone resorption from 2- to 3-fold of the original awl diameter, peri-hole bone resorption was only detected in 3 out 30 microfracture holes (10%) in the minipig model and in 7 out of 30 microfracture holes (23%) in the sheep model. When lowering the threshold for peri-hole bone resorption from 2- to 1.5-fold of the original awl diameter, peri-hole bone resorption was detected in 30 out of 30 microfracture holes (100%) in the minipig model and in 21 out of 30 microfracture holes (70%) in the sheep model (Fig. 4B).

#### Analysis of the threshold for defining subchondral bone cysts

Likewise, when subchondral bone cysts were defined as suggested above (Hd_**2**_ larger than the triple diameter of the microfracture awl), this pathological condition was detected in 1 out of 30 microfracture holes (3%) in the minipig model and 5 out of 30 microfracture holes (17%) in the sheep model (Fig. 4C). If the threshold was elevated from 3- to 4-fold of the original awl diameter, no cyst was detected in the minipig model and only 1 subchondral bone cyst was detected in the sheep model (3%). When lowering the threshold from 3- to 2-fold of the original awl diameter, subchondral bone cysts were still detected in 1 out of 30 microfracture holes (3%) in the minipig model and but in 16 out of 30 microfracture holes (53%) in the sheep model (Fig. 4C).

### Inter-rater reliability of the algorithm for evaluation of the subchondral bone alterations

The calculation of inter-rater reliability of the novel algorithm revealed a total number of observed agreements of 27/30, corresponding to 90% of all observations. This resulted in a Cohen’s kappa of 0.874.

### Comparison of the present algorithm with the most comprehensive existing scoring system

The micro-CT datasets of defects from both animal models were then evaluated with the most comprehensive existing categorical scoring system described by Chen *et al*. (Supplementary Table S1)^12^.

#### Intra-lesional osteophytes

Applying the Chen score yielded point values ranging between 0 (>90% of defect width and length) and 2 (<5% of defect width and length). Applying the present method enabled a separate analysis of each osteophyte within defects containing multiple osteophytes (Supplementary Table S3). In addition, more detailed information for each osteophyte was provided by the present algorithm such as maximal height (0.312 ± 0.119 mm in minipigs; 0.258 ± 0.111 mm in sheep), maximal width (0.522 ± 0.216 mm in minipigs; 1.175 ± 0.837 mm in sheep), and maximal 2D area (0.204 ± 0.184 mm^2^ in minipigs; 0.247 ± 0.167 mm^2^ in sheep).

#### Discrimination between residual microfracture holes, peri-hole bone resorption, and subchondral bone cysts

When applying the Chen score to both datasets of defects, a precise discrimination between residual holes and peri-hole bone resorption could not be achieved (Fig. 4Ai–iv). Moreover, coexistence of both residual microfracture holes and subchondral bone cysts in a single defect was detected in the 5 defects in sheep (Fig. 4Av–viii), in which the Chen score did not allow allocating them to a single point value (Supplementary Table S2). In contrast, the new scoring system permitted an objective and reproducible discrimination between both pathologies (Fig. 5Bi,ii,iv,v). Specifically, residual microfracture holes were detected in 7 out of 30 original microfracture holes in the minipig model and in 8 out of 30 original microfracture holes in the sheep model. Subchondral bone cysts were observed in 1 out of 30 microfracture holes in the minipig model and in 5 out of 30 microfracture holes in the sheep model.

For the quantitative evaluation of bone resorption in both animal models, the Chen score solely allows for a subjective and general estimation of the severity of bone resorption within an entire defect (Supplementary Table S4 and Fig. 5Aii,iv) without discernment between bone resorption and residual microfracture holes (Fig. 4Ai–iv). On the contrary, the present continuous method is designed to yield an easy and objective evaluation of bone resorption around each original microfracture hole based on quantitative data (Fig. 5Biii,vi). Peri-hole bone resorption was detected in 22 out of 30 microfracture holes in the minipig model and in 17 out of 30 microfracture holes in the sheep model.

## Discussion

The key contributions of this paper are (1) to give an overview of the analytic methods currently described in the PubMed literature to examine subchondral bone changes, (2) to provide clear definitions of the four types of subchondral bone changes and to develop a novel algorithm allowing for analysing and distinguishing these subchondral bone changes based on micro-CT images, (3) to evaluate this new algorithm in datasets obtained from two large animal models, and (4) to compare it with the best existing semi-quantitative scoring system.

Here, the term “intra-lesional osteophyte” is defined as an osseous structure above the projected cement line. In a rabbit model following microfracture, Chen *et al*. defined an intra-lesional osteophyte as bone overgrowth above the projected tidemark in micro-CT image^12^. This definition does not reflect the fact that the calcified cartilage (and tidemark) is usually debrided to expose the subchondral bone plate in the standard microfracture procedure^4^. Similarly, Shive *et al*. reported a limited incidence of intra-lesional osteophytes in patients after microfracture treatment by 3D quantitative MRI. The morphology of the intra-lesional osteophyte was calculated using the ratio of intra-lesional bone volume to the entire debrided lesion volume at one month postoperatively^19^. However, the change of this relative intra-lesional bone volume (termed “percentage of intra-lesional osteophyte”) might be the result of other types of subchondral bone changes, such as peri-hole bone resorption or cyst formation^5^, rather than an intra-lesional osteophyte.

A key improvement of the suggested algorithm is that it allows for a specific, objective, and reproducible analysis of each single microfracture hole. In practice, it is very common to obtain a micro-CT image with a suspected bone bridge within the defect. The relative height of the suspected bone bridge is a novel parameter for defining the possible number of intra-lesional microfracture holes, allowing for an assessment of each microfracture hole separately. Clinically, the microfracture hole should be established perpendicular to bone surface and independent to neighbouring holes. Therefore, the individual analysis for each microfracture hole provides more detailed information of the entire microfracture procedure.

Defining the horizontal diameter of the opening of the microfracture hole is useful to differentiate between residual microfracture holes and peri-hole bone resorption. To identify a residual hole, the double diameter of the microfracture awl is chosen as the upper threshold value. This value takes into consideration a possible deflection of the awl during its impaction into the subchondral bone compartment, which, in theory, may cause an expansion of the hole. This recommended threshold for residual holes is consistent with the previous study of Hoemann *et al*., showing that the maximal apical diameter of a microfracture hole (2.0 mm) was less than the double diameter of the original microfracture awl (1.1 mm)^20^. Additionally, based on the aforementioned descriptive analysis of peri-hole bone resorption in two different animal models, it is also suggested that the maximum diameter of the residual hole at its apical opening (Hd_1_) should be less than the double of the original awl diameter in order to fulfil the conditions of being a residual hole. If Hd_1_ is larger than the double of awl diameter, a peri-hole bone resorption is defined.

For practicability of this algorithm, 3-fold the diameter of the original awl at the level of half of the penetration depth was chosen as threshold for subchondral bone cysts. This resulted in the identification of cysts in 3% of the defects in the minipigs and 17% of the defects in sheep, respectively. When this threshold value was only slightly shifted, extremely lower (3% for 4-fold awl diameter) and higher (53% for 2-fold awl diameter) incidences were calculated, most likely not in line with the biological situation. Therefore, elevating or lowering the recommended threshold value is not suggested, as this might result in either overestimation or underestimation of the incidence of these subchondral bone alterations.

The high reliability of the present algorithm has been verified with the calculation of inter-rater agreement (90% agreement) and Cohen’s kappa (kappa = 0.874). In the independent blinded evaluations by two observers using the present algorithm, inter-rater disagreement only existed in 3 out of the total 30 randomly selected representative defects from two animal models. These data prove that the algorithm is highly reliable, reproducible, and valid for analysis the subchondral bone alterations in different animal models.

The enhanced precision of this tailored algorithm was demonstrated by the comparison with the most comprehensive Chen score^12^. Compared with the semi-quantitative evaluation of the Chen score, the present algorithm uses the diameter of the microfracture awl as a stable reference and provides more precise definition of each pattern of subchondral bone changes. Most importantly, it avoids the potential pitfalls of the Chen score that does not differentiate between residual microfracture holes, peri-hole bone resorption, and subchondral bone cysts. Additionally, this algorithm enables the objective and reproductive analysis of subchondral bone change around each original microfracture hole.

The present algorithm has some limitations. First, owing to the specimen processing, the thresholds for subchondral bone cyst and peri-hole bone resorption were not tested by other modalities, such as inter-group comparison of standardized osteoclast histomorphometry. However, the present analysis in two large animal models proved the practicability and feasibility of the recommended thresholds. Secondly, the general upward migration of the subchondral bone plate–a possible independent type of subchondral bone alterations^5^–was not detected in the datasets from the minipig and sheep models, therefore it was not included into the present algorithm. Finally, the different location of the defects in the two animal models (trochlear groove *versus* femoral condyle) may weaken the algorithm. As cartilage repair differs in the sheep trochlea compared with the medial femoral condyle^21^, the suggested thresholds for bone cyst and peri-hole resorption may need to be adapted accordingly if the extent and pattern of subchondral bone alterations are similarly topographically dependent.

In the past decades, the subchondral bone has been increasingly recognized as being fundamentally important for the clinical success of articular cartilage repair^5^,^18^,^20^,^22^,^23^,^24^,^25^. Microfracture, representing the clinically most relevant first-line treatment option for small chondral lesions, has been extensively evaluated in patients with numerous outcome parameters and at various follow-up periods^1^,^3^,^8^,^9^,^25^,^26^. Our still insufficient understanding of subchondral bone repair after microfracture treatment is a strong motivation to further investigate the mechanisms of postoperative osteochondral remodelling in animal models. The current algorithm may further help standardising the analysis of subchondral bone changes following articular cartilage repair. A deeper understanding of the complex role of the subchondral bone is beneficial to evaluate the outcome of current treatment strategies and to further translate cell- or biomaterial-based techniques to the clinic. The proposed algorithm could conveniently be adapted to identify and analyse subchondral bone changes such as intra-lesional osteophytes, residual microfracture holes, peri-hole bone resorption, and subchondral bone cysts also in conventional CT or MRI protocols. This algorithm possesses a high inter-rater reliability and may therefore serve as a basis for a more practical, objective, and reproducible evaluation of postoperative subchondral bone alterations in both animal models and patients in the future.

## Methods

### Systematic review of current methods for analysing subchondral bone changes following microfracture treatment

For summarizing the current methods in evaluating the postoperative status of the subchondral bone, a systematic review of subchondral bone changes following microfracture treatment in animal models was performed according to Preferred Reporting Items for Systematic Reviews and Meta-Analyses (PRISMA) standards and a PRISMA checklist^27^. The PubMed database was searched from 1950 through April 15, 2016. Inclusion criteria for the search included English language, animal studies with microfracture treatment, and evaluation of the subchondral bone.

### Micro-CT imaging of the subchondral bone following microfracture treatment

In these studies, full-thickness chondral defects were created in the femoral trochlea of adult minipigs (n = 5 animals, 2 defects per animal; age: 18–22 months; average body weight: 28.9–41.7 kg) and the medial femoral condyle of adult sheep (n = 5 animals, 1 defect per animal; age: 26–42 months; average body weight: 72.7–81.5 kg). The border of the cartilage defects was outlined with a circular (4-mm diameter in minipigs) or rectangular punch (4 × 8 mm in sheep). Next, the hyaline cartilage was removed with a curette (Fig. 3Ai) to create full-thickness chondral defects that were circular in minipigs (Fig. 3Aii) and rectangular in sheep. The calcified cartilage layer was meticulously removed to expose the subchondral bone plate (Fig. 3Aiii). Microfracture holes were created in the subchondral bone plate using a microfracture awl in a standardized fashion. Three holes per defect in minipigs (Fig. 3Aiv,v) and six holes per defect in sheep were introduced. Holes were always orientated perpendicular to the bone surface and distributed evenly within the defects (resulting in a similar distance between holes)^4^. The applied microfracture awl (Fig. 3Avi) consists of a conical head and a cylindrical body (1.2 mm diameter). A penetration stop ensured a standardized penetration depth^25^.

Osteochondral specimens containing the defects were scanned along the axis of each microfracture hole in a micro-CT scanner (Skyscan 1172, Skyscan, Kontich, Belgium) as previously described^18^. The device possesses a moveable 10-MP camera and an X-ray tube (<5 μm spot; Hamamatsu, Hamamatsu City, Japan), allowing for a maximal nominal resolution below 0.8 μm.

The X-ray tube voltage was 70 kV, and the current was 140 μA. All specimens were scanned within 70% ethanol at a spatial resolution of 15 μm. X-ray projections were obtained at 0.4° intervals with 1770 ms exposure time and a combined 0.5 mm aluminum/copper filter interposed. Ring artifact correction, frame averaging, and random movement were engaged (4, 3, and 15, respectively, no units). The reconstructed dataset was segmented with a modified Feldkamp cone-beam algorithm^28^.

In order to mimic bone as closely as possible, the thresholding levels of gray values (range: 89–255) were set for segmentation of binary images. To express gray values as bone mineral density (BMD), calcium hydroxyapatite (CaHA) phantom rods immersed in 70% ethanol with known BMD values (250 and 750 mg CaHA/cm^3^) were employed for calibration.

Theoretically, in the present minipig model (3 holes per defect), only two kinds of micro-CT image could be obtained: the micro-CT image displays either one single hole (Fig. 3Bi–iii) or two microfracture holes (Fig. 3Biv–vi).

### Algorithm for analysing of subchondral bone changes

The precise definition of each type of subchondral bone change was provided and a novel micro-CT based algorithm was proposed for evaluating these subchondral bone changes (Fig. 1). This algorithm was then applied and validated with the entire above-mentioned micro-CT datasets from both minipig and sheep models.

### Inter-rater reliability of the algorithm

To test the reliability of this algorithm, 30 representative image sets with subchondral bone alterations from the sheep and minipig models were chosen by observer one (PO) and blinded for the following evaluation. Observers two and three independently scored the images using the novel algorithm. After completion, the number of observed agreements and Cohen’s kappa were calculated^29^.

### Comparison of the algorithm with the most comprehensive existing method

To evaluate the quality of the new algorithm, the datasets of defects from both animal models were evaluated applying the most comprehensive existing categorical scoring system (Supplementary Table S1). The results of the best existing method were compared with that of the present algorithm.

### Statistical analysis

Descriptive data are expressed as mean value ± standard deviation. Units of the SI system are used where appropriate. Cohen’s (unweighted) kappa was used to evaluate inter-rater agreement of nominal scaled values between two independent observers^29^. Calculations were performed using Microsoft Excel 2007 (Microsoft, Redmond, WA, USA) and the GraphPadQuickCalcs Website: http://graphpad.com/quickcalcs/kappa1 (accessed December 16, 2015).

## Additional Information

**How to cite this article**: Gao, L. *et al*. A novel algorithm for a precise analysis of subchondral bone alterations. *Sci. Rep.* **6**, 32982; doi: 10.1038/srep32982 (2016).

## Supplementary Material

Supplementary Information

## Figures and Tables

**Table 1:** Overview of the four major types of subchondral bone changes after microfracture treatment and corresponding evaluation methods in animal models identified in the PubMed database.

Pathology	Procedure	Species	Animal numbers	Follow-up	Evaluation method	Criteria of evaluation	Reference
Intra-lesional osteophyte	Microfracture; intraarticular injection of bone marrow stem cells	Horse	12	10 months	MRI	Scaled score ranging from 0 (normal) to 4 (severe)	Mcllwraith *et al*.^17^
Intra-lesional osteophyte	Microfracture	Rabbit	16	3 months	Micro-CT	Scaled score based on the relative size of the osteophyte	Chen *et al*.^12^
Residual microfracture hole	Microfracture; chitosan-glycerol phosphate/ blood	Sheep	8	2.5 hours	Histology	Qualitative description based on histological images	Hoemann *et al*.^16^
Residual microfracture hole	Microfracture	Rabbit	2	1 day	Micro-CT	Semi-quantitative description based on micro-CT and histological images	Chen *et al*.^30^
Residual microfracture hole	Microfracture	Rabbit	16	3 months	Micro-CT	Scaled score based on the number of holes in micro-CT images	Chen *et al*.^12^
Peri-hole bone resorption	Microfracture; chitosan-glycerol phosphate/ blood	Sheep	8	6 months	Histomorphometry	Quantitative description based on the percentage of defect filling	Hoemann *et al*.^16^
Peri-hole bone resorption	Microfracture	Monkey	12	1.5 months	Histology	Qualitative description based on histological images	Gill *et al*.^15^
Peri-hole bone resorption	Microfracture	Rabbit	16	3 months	Micro-CT	Scaled score ranging from 0 (significant) to 2 (little or none)	Chen *et al*.^12^
Subchondral bone cyst	Microfracture	Horse	5	4 months	Plain radiograph	Scaled score ranging from 1 (flat subcentral bone) to 4 (large lytic area deep to subchondral bone)	Frisbie *et al*.^14^
Subchondral bone cyst	Microfracture; chitosan-glycerol phosphate/ blood	Sheep	8	6 months	Histomorphometry	Scaled score ranging from 0 to 4 based on the diameter of fibrous white tissue or tissue voids	Hoemann *et al*.^16^
Subchondral bone cyst	Microfracture	Horse	12	4 months	Plain radiograph; MRI	Scaled score ranging from 0 (normal) to 3 (severe)	Frisbie *et al*.^13^
Subchondral bone cyst	Microfracture	Rabbit	8	3 months	Histomorphometry; Micro-CT	Quantitative evaluation and description based on micro-CT and histological images	Chen *et al*.^12^
