# Supplementary material for: A novel algorithm for a precise analysis of subchondral bone alterations
Source: Sci Rep. 2016 Sep 6;6:32982. doi: 10.1038/srep32982 (PMC5011758; doi:10.1038/srep32982)
Supplement: Supplementary Information [file srep32982-s1.pdf]

Supplementary Materials

A novel algorithm for a precise analysis of subchondral bone alterations

Liang Gao[1], Patrick Orth[1, 2], Lars K. H. Goebel[1, 2], Magali Cucchiarini[1], Henning Madry[1,2,*]

[1]Center of Experimental Orthopaedics, Saarland University, Homburg, Germany.

[2] Department of Orthopaedic Surgery, Saarland University Medical Center, Homburg, Germany

[*]Corresponding author

E-mail: henning.madry@uks.eu

List of Supplementary Materials

Supplementary Figure 1. Flowchart of the literature searching in the PubMed database.

Supplementary Table S1. The Chen scoring system for a semi-quantitative assessment of the presence of intra-lesional osteophyte (termed "bone overgrowth above projected tidemark").

Supplementary Table S2. Semi-quantitative estimation of residual microfracture holes and subchondral bone cysts in the minipig and sheep models applying the Chen score.

Supplementary Table S3. Semi-quantitative estimation of intra-lesional osteophytes in the minipig and sheep models applying the Chen score and the present algorithm.

Supplementary Table S4. Semi-quantitative Evaluation of bone resorption in the minipig and sheep models applying the Chen score.

Supplementary Figure 1. Flowchart of the literature searching in the PubMed database.

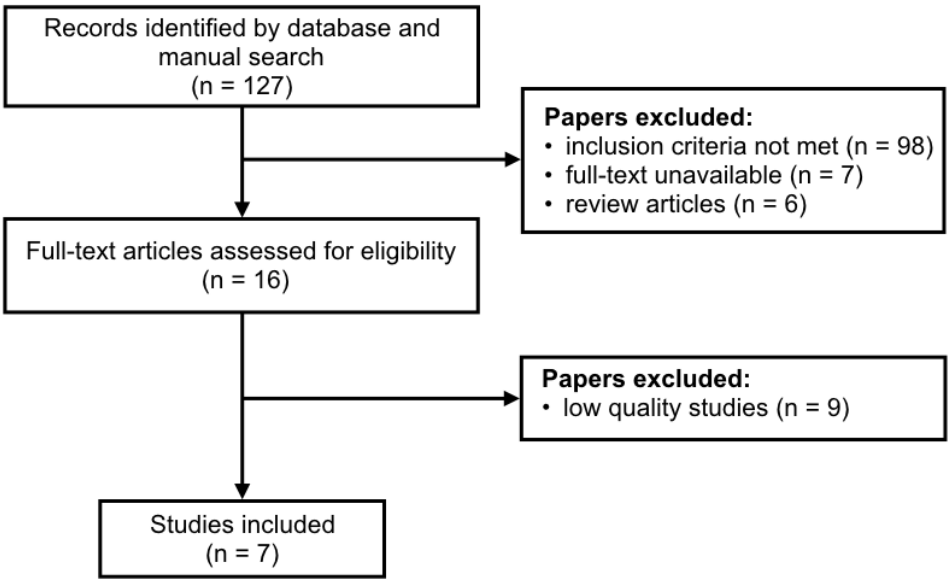

Supplementary Table S1. The Chen scoring system for a semi-quantitative assessment of the presence of intra-lesional osteophyte (termed "bone overgrowth above projected tidemark"), residual microfracture holes or subchondral bone cyst formation, and bone resorption. Only the relevant variables 1-3 from the original Chen score are shown[12].

**Variables**	**Point scale**
**Bone overgrowth above projected tidemark**	
Proud throughout (exhibited in > 90% of defect width and length)	0
Proud throughout (exhibited in 5% to 90% of defect width and length)	1
Little or none (exhibited in < 5% of defect width and length)	2
**Presence of residual holes or cyst**	
Subchondral bone cyst or large merged hole	0
3 or more holes	1
2 holes	2
1 hole	3
No visible holes	4
**Bone resorption**	
Significant	0
Moderate	1
Little or none	2

Supplementary Table S2. Semi-quantitative estimation of residual microfracture holes and subchondral bone cysts in the minipig and sheep models applying the Chen score. Note that the scoring system described by Chen *et al*. can only subjectively discriminate residual holes from subchondral bone cysts[12]. No definite point scale can be given in specific defects with both suspected residual holes and suspected cysts inside. Discrimination between residual hole and peri-hole resorption is also not feasible with the Chen score. *point scale for residual microfracture holes; $^{\#}$ point scale for subchondral bone cysts.

	**Chen score**			
**Animal type**	**Animal number**	**Number of residual holes**	**Number of subchondral bone cysts**	**Point scale**
Minipig	1	2	1	$0^{\#}/2^{*}$
Minipig	2	1	0	3^{*}
Minipig	3	2	1	$0^{\#}/2^{*}$
Minipig	4	1	0	3^{*}
Minipig	5	2	0	2^{*}
Minipig	6	2	1	$0^{\#}/2^{*}$
Minipig	7	1	0	3^{*}
Minipig	8	2	1	$0^{\#}/2^{*}$
Minipig	9	1	0	3^{*}
Minipig	10	1	0	3^{*}
Sheep	1	1	4	$0^{\#}/3^{*}$
Sheep	2	2	2	$0^{\#}/2^{*}$
Sheep	3	4	1	$0^{\#}/1^{*}$
Sheep	4	2	1	$0^{\#}/2^{*}$

Sheep	5	1	1	0[#]/3[*]

Supplementary Table S3. Semi-quantitative estimation of intra-lesional osteophytes in the minipig and sheep models applying the Chen score and the present algorithm. Note that the scoring system described by Chen *et al*. evaluates the relative size of osteophyte (termed "bone overgrowth above the projected tidemark") to the defect[12]. It provides a general description of the entire defect while it is not practical in defects with multiple osteophytes. The present algorithm can quantitatively estimate each intra-lesional osteophyte separately and provide details of each osteophyte.

	**Chen score**		**Present algorithm**			
**Animal type**	**Animal number**	**Point scale**	**Number of osteophytes**	**Maximal height (mm)**	**Maximal width (mm)**	**Maximal 2D area (mm^2)**
Minipig	1	1	1	0.446	0.762	0.416
Minipig	2	1	1	0.219	0.461	0.110
Minipig	3	1	1	0.271	0.342	0.086
Minipig	4	2	0	-	-	-
Minipig	5	2	0	-	-	-
Minipig	6	2	0	-	-	-
Minipig	7	2	0	-	-	-
Minipig	8	2	0	-	-	-
Minipig	9	2	0	-	-	-
Minipig	10	2	0	-	-	-
Sheep	1	1	3	0.104	0.611	0.181
				0.297	0.404	0.151
				0.189	0.745	0.093

Sheep	2	1	1	0.283	2.328	0.522
Sheep	3	1	1	0.242	1.788	0.381
Sheep	4	1	1	0.258	1.044	0.247
Sheep	5	2	0	-	-	-

Supplementary Table S4. Semi-quantitative Evaluation of bone resorption in the minipig and sheep models applying the Chen score. Note that the scoring system described by Chen *et al*. provides a subjective and general estimation of the severity of bone resorption of the entire defect[12], but it can not differentiate bone resorption from bone cyst and provide evaluation for each microfracture hole.

	**Chen score**		
**Animal type**	**Animal number**	**Severity of bone resorption**	**Point scale**
Minipig	1	Significant	0
Minipig	2	Moderate	1
Minipig	3	Moderate	1
Minipig	4	Moderate	1
Minipig	5	Significant	0
Minipig	6	Significant	0
Minipig	7	Significant	0
Minipig	8	Significant	0
Minipig	9	Moderate	1
Minipig	10	Significant	0
Sheep	1	Moderate	1
Sheep	2	Little or none	2
Sheep	3	Moderate	1
Sheep	4	Moderate	1
Sheep	5	Little or none	2
